# Prediction of Pubertal Mandibular Growth in Males with Class II Malocclusion by Utilizing Machine Learning

**DOI:** 10.3390/diagnostics13162713

**Published:** 2023-08-21

**Authors:** Grant Zakhar, Samir Hazime, George Eckert, Ariel Wong, Sarkhan Badirli, Hakan Turkkahraman

**Affiliations:** 1 Department of Orthodontics and Oral Facial Genetics, Indiana University School of Dentistry, Indianapolis, IN 46202, USA; gzakhar@iu.edu (G.Z.); arwong@iu.edu (A.W.); 2 Indiana University School of Dentistry, Indianapolis, IN 46202, USA; shazime@iu.edu; 3 Department of Biostatistics and Health Data Science, Indiana University School of Medicine, Indianapolis, IN 46202, USA; geckert@iu.edu; 4 Eli Lily & Company, Indianapolis, IN 46285, USA; s.badirli@gmail.com

**Keywords:** artificial intelligence, machine learning, mandibular growth, growth prediction

## Abstract

The goal of this study was to create a novel machine learning (ML) model that can predict the magnitude and direction of pubertal mandibular growth in males with Class II malocclusion. Lateral cephalometric radiographs of 123 males at three time points (T1: 12; T2: 14; T3: 16 years old) were collected from an online database of longitudinal growth studies. Each radiograph was traced, and seven different ML models were trained using 38 data points obtained from 92 subjects. Thirty-one subjects were used as the test group to predict the post-pubertal mandibular length and *y*-axis, using input data from T1 and T2 combined (2 year prediction), and T1 alone (4 year prediction). Mean absolute errors (MAEs) were used to evaluate the accuracy of each model. For all ML methods tested using the 2 year prediction, the MAEs for post-pubertal mandibular length ranged from 2.11–6.07 mm to 0.85–2.74° for the *y*-axis. For all ML methods tested with 4 year prediction, the MAEs for post-pubertal mandibular length ranged from 2.32–5.28 mm to 1.25–1.72° for the *y*-axis. Besides its initial length, the most predictive factors for mandibular length were found to be chronological age, upper and lower face heights, upper and lower incisor positions, and inclinations. For the *y*-axis, the most predictive factors were found to be *y*-axis at earlier time points, SN-MP, SN-Pog, SNB, and SNA. Although the potential of ML techniques to accurately forecast future mandibular growth in Class II cases is promising, a requirement for more substantial sample sizes exists to further enhance the precision of these predictions.

## 1. Introduction

The post-natal growth of the human mandible holds great significance within the field of orthodontics, as it boasts the highest growth potential among craniofacial structures [1]. The majority of mandibular growth takes place during adolescence, which coincides with the common treatment period for orthodontic patients [2]. Normal mandibular growth is typically observed in Class I patients, where the development of the mandible proceeds without significant deviations or abnormalities. Unanticipated mandibular growth can notably influence outcomes, particularly in Class III patients where excessive growth poses challenges. Conversely, in Class II cases, there is often a prominent deficiency in mandibular growth, characterized by insufficient horizontal and/or vertical development [3,4]. This deficiency limits the potential for self-correction without the intervention of orthodontic treatment [5]. To address these challenges, growth modification therapies have been utilized for decades to enhance mandibular growth while concurrently restricting maxillary growth [6]. If orthodontists had the ability to accurately predict mandibular growth, this would hold immense value as it would enable clinicians to anticipate and plan orthodontic treatment effectively, allowing for timely interventions to guide and optimize mandibular development, resulting in improved treatment outcomes and long-term stability.

In the 1960s, Bjork sought to understand normal growth variation by placing metallic implants in the jaws of developing children [7,8,9]. His approach aimed to unravel the mysteries of mandibular growth, enabling the prediction of both the magnitude and direction of growth with greater precision. In his research, Bjork deduced that the mandible exhibits a predominant downward and forward growth pattern, with the condyle serving as the primary site of substantial growth [9]. Skieller et al. made a significant contribution to mandibular growth prediction using longitudinal studies and cephalometric analysis. Their research focused on establishing growth patterns predictions based on intermolar angle, the shape of the lower border of the mandible, the inclination of the symphysis, and mandibular inclination [10]. Though their methods were thought to have high accuracy, subsequent clinical evaluation exhibited notable inaccuracy and limitations [11]. Thereafter, Ricketts and his colleagues [12,13] proposed an arcial prediction method, which exhibited promising clinical utility when subjected to preliminary testing with a limited sample size, subsequently earning recognition as a prediction method currently integrated into the Dolphin Imaging 11.0 Software. Ultimately, predictions derived from anatomical structures have demonstrated inconsistent accuracy.

In the pursuit of more accurate predictions, several mathematical models have been developed. Rudolph et al. incorporated the Bayes theorem and Gaussian distribution to develop a statistical model that predicted mandibular growth based on observed variables and their probabilistic relationships [14]. Their approach leveraged both prior knowledge and the data at hand to estimate and predict mandibular growth. However, this method demonstrated a prediction accuracy of only 82%. Buschang et al. developed a mathematical model that involved comparing the average yearly growth velocities with a population-based growth curve [15]. Their findings demonstrated a prediction accuracy of 76%; the researchers acknowledged the presence of bias due to anticipated growth variations that could not be fully accounted for by the prediction methods employed. In 2021, Jiménez-Silva et al. conducted a systematic review investigating Class II mandibular growth and reached a significant conclusion, highlighting the overall low-to-moderate methodological quality of existing predictors and underscoring the pressing need for reliable prediction methods [16]. Numerous studies were found to possess an elevated risk of bias and employed broad sample selections, further emphasizing the need for rigorous investigation. As a result, this systematic review advocates for the implementation of a meticulously designed longitudinal cohort study based on lateral cephalometric radiographs, which adhere to stringent quality standards, to address this research gap and provide more accurate predictions. Despite concerted efforts, it remains challenging for human-made models to comprehensively account for the intricate and multifaceted variations in human beings.

Walker was one of the first in the field of orthodontics to postulate and conduct mandibular predictions using computer software [17]. Since then, technology has advanced so significantly that artificial intelligence (AI) and machine learning (ML) have been utilized in almost every aspect of our life. AI is the development of computer systems capable of performing tasks that normally require human intelligence [18]. Within AI, ML utilizes a set of inputs and outputs to create an algorithm to process the data and correctly predict the output [19]. AI and ML have been utilized for several tasks in orthodontics, such as for automated cephalometric analyses [20,21,22,23,24], predicting extraction vs. non-extraction treatment decisions [25,26,27,28,29,30,31,32,33], predicting orthodontic extraction patterns [34], determining the need for surgery in Class III patients [35], and growth assessment [36,37,38,39,40,41,42,43,44]. However, little research has been conducted on the use of AI to predict mandibular growth. Niño-Sandoval et al. utilized automated learning techniques to predict mandibular morphology in Class I, II, and III patients [45]. This study used the coordinates of craniofacial landmarks as variables for Artificial Neural Networks and Support Vector Regression (SVR) to predict morphological outcomes. This research yielded exceptional predictability, showcasing the remarkable ability of AI to accurately forecast jaw morphology. The same group of researchers used AI to classify skeletal patterns through craniomaxillary variables selected from the mandible for forensic use [46]. This resulted in 74% accuracy in correctly predicting the skeletal patterns. In an unpublished master thesis, Jiwa et al. employed deep learning techniques to construct an algorithm for mandibular growth prediction [47]. Their approach involved utilizing predictions based on the X and Y coordinates of 17 mandibular landmarks on selected cephalograms and comparing them with Rickett’s growth prediction. However, this proved to be generally inaccurate, highlighting the necessity for larger and more targeted sample populations to enhance the predictive capabilities. Recently, Wood et al. utilized 39 linear and angular measurements from lateral cephalograms to predict mandibular growth in untreated Class I male patients [48]. This study employed seven distinct ML algorithms to analyze the measurements, predict the magnitude and direction of the mandible, and subsequently compare the results to the final cephalogram of each patient. They were able to predict mandibular growth within 3 mm and *y*-axis within 1°.

To the best of our knowledge, there has been no previous research investigating the application of ML for accurately predicting both the magnitude and direction of mandibular growth in adolescent males presenting with a Class II malocclusion during the circumpubertal period. As mentioned earlier, existing predictions of mandibular growth have often lacked the desired precision and accuracy. Achieving a breakthrough in the field of orthodontics through such predictions would mark a significant advancement. By gaining the capability to forecast mandibular growth in Class II patients, we could determine the optimal timing to initiate treatment, assess the need for any growth modification, and provide patients with the most effective and exceptional treatment plans possible, which is of paramount importance. The goal of this investigation is to develop an ML algorithm that can reliably and accurately forecast the magnitude and direction of mandibular growth within this specific patient subgroup.

## 2. Materials and Methods

### 2.1. Ethics

This retrospective study was approved as a non-human subjects research (NHSR) by the Institutional Review Board (IRB) of Indiana University Human Research Protection Program (HRPP) (Protocol #14987).

### 2.2. Study Sample

The sample of this study consisted of digital cephalometric radiographs from subjects in the American Association of Orthodontists Foundation (AAOF) Craniofacial Legacy Collection, which includes data from the Bolton Brush Growth, Burlington Growth, Denver Growth, Fels Longitudinal, Forsyth Twin, Iowa Growth, Matthews Growth, Michigan Growth, and Oregon Growth studies [49]. The inclusion criteria consisted of males with Class II malocclusion or an ANB > 3.5 with pre-pubertal (T1) (mean age ± SD: 12.0 ± 0.29 years), pubertal (T2) (mean age ± SD: 14.1 ± 0.27 years), and post-pubertal (T3) (mean age ± SD: 15.9 ± 0.48 years) cephalograms. Subjects with craniofacial anomalies, apparent skeletal asymmetries, missing teeth (excluding third molars), missing cephalometric records, or lateral cephalograms lacking necessary structures were excluded from this study. A total of 123 cases met the inclusion criteria and were selected for this study.

### 2.3. Sample Size Justification

The study used 92 of the cases for training and the remaining 31 for the testing set. With this sample size, the 95% confidence interval for the intra-class correlation coefficients (ICCs) had a width of 0.28, extending from 0.62 to 0.90, if the ICC was 0.80; higher ICCs had shorter confidence interval widths.

### 2.4. Data Collection

**Figure 1:**
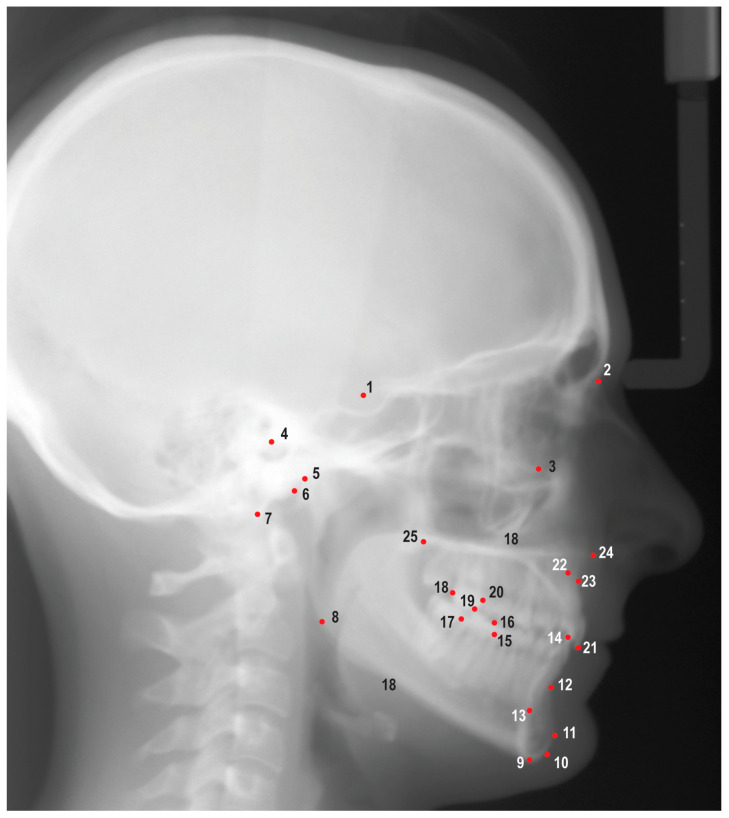
Cephalometric landmarks used in this study: 1. Sella (S), 2. nasion (N), 3. orbitale (Or), 4. porion (Po), 5. condylion (Co), 6. articulare (Ar), 7. basion (Ba), 8. gonion (Go), 9. menton (Me), 10. gnathion (Gn), 11. pogonion (Pog), 12. B point (B), 13. lower incisor root apex (L1a), 14. lower incisor incisal edge (L1i), 15. mesial of lower first molar (L6m), 16. mesiobuccal cusp of lower first molar (L6mb), 17. distal of lower first molar (L6d), 18. distal of upper first molar (U6d), 19. mesiobuccal cusp of upper first molar (U6mb), 20. mesial of upper first molar (U6m), 21. upper incisor incisal edge (U1i), 22. upper incisor root apex (U1a), 23. A point (A), 24. anterior nasal spine (ANS), and 25. posterior nasal spine (PNS).

Images obtained from the AAOF collection were then imported into Dolphin Imaging V. 11.95 (Dolphin Imaging and Management Solutions, Chatsworth, CA, USA) for further analyses. A solitary investigator (G.Z.) identified and annotated 25 hard tissue landmarks on each image (Figure 1). This process enabled the calculation of 38 linear and angular measurements, which were subsequently utilized as hyperparameters for the model (Table S1). Several cephalograms did not show adequate soft tissue; therefore, soft tissue landmarks and associated cephalometric measurements were not included in the study.

The AAOF provided dots-per-inch (DPI) calibration for measurements; however, when magnification discrepancies were detected, images were printed at a 1:1 scale, and ruler length was verified for accuracy, whereafter the digital ruler was employed to recalibrate measurements. Demographic and cephalometric data were then compiled and stored in a secure cloud service (OneDrive, Microsoft Co., Redmond, WA, USA). For the intra-examiner repeatability assessment, a research randomizer was utilized to randomly choose 20 images for retracing. ICCs were utilized to evaluate the measurements’ repeatability.

### 2.5. Algorithm Training and Testing

**Figure 2:**
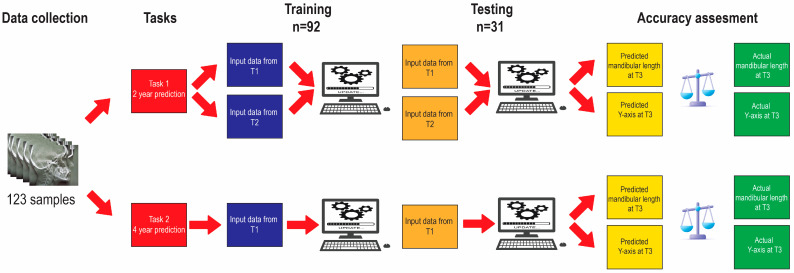
Algorithm training and testing workflow.

The dataset was randomly separated into 75% training data for training the model and 25% testing data for testing the model. The training set’s purpose was to impart knowledge to the ML models so that they could accurately forecast the post-pubertal mandibular length and *y*-axis. To this end, input data obtained from both T1 and T2 were used for a 2 year prediction, whereas input data from only T1 were utilized for a 4 year prediction (Figure 2).

Six fundamental traditional regression techniques, XGBoost, Random Forest, Lasso, Ridge, Linear Regression, and Support Vector Regression (SVR), along with a Multilayer Perceptron (MLP) regressor were used to ensure the robustness of our investigation. To investigate possible linear associations, we employed Linear Regression utilizing the least squares method, along with L1 (Lasso) and L2 (Ridge) regularizers. The Linear Regression technique, which is a venerable statistical tool, facilitates approximations for problems in which the number of equations exceeds the number of unknowns. This approach is particularly adept at unearthing linear relationships that might underlie the data. For data that deviated from the linear trajectory, we utilized non-linear methodologies such as kernel-based SVR, tree-based algorithms such as XGBoost and Random Forest, and the MLP regressor. Random Forest, an ensemble of decision trees, was employed to mitigate the disparity between predicted and actual dependent variables, as well as to minimize overfitting, especially given the limitations of our constrained training dataset. All experiments were conducted in Spyder 4.1.5, utilizing the programming language Python 3.7.9 (Python Software Foundation, Fredricksburgh, VA, USA). To carry out the experiments, the following packages were used: sklearn version 1.0.2 (NumFOCUS, Austin, TX, USA) for least squares, Ridge, Lasso, and Random Forest; XGBoost version 1.5.0 (DMLC, Seattle, WA, USA) for XGBoost; and Keras version 2.4.0 (Keras, Mountain View, CA, USA) in the TensorFlow version 2.4.3 (Keras, Mountain View, CA, USA) platform for the neural network.

### 2.6. Statistical Analysis

The mean absolute error (MAE), root mean square error (RMSE), mean error (ME), ICCs, and Bland–Altman plots were calculated for each technique to evaluate the agreement between the predicted and actual outcome measurements. The accuracy percentage of the methods was calculated using the formula (1 − (MAE/Actual Value) × 100). The directional and absolute differences between the predicted and actual measurements were calculated and compared between models using analysis of variance (ANOVA), with random effects to correlate data from the same patient. Paired t-tests were used to test for a significant mean directional difference between predicted and actual measurements. A two-sided 5% significance level was used for all the tests. All analyses were performed using SAS version 9.4 (SAS Institute, Inc., Cary, NC, USA).

## 3. Results

### 3.1. Reliability Analysis

The results of the reliability analysis are presented in Table S2. Most variables exhibited excellent repeatability (ICCs > 0.90), with the remainder having good repeatability (0.75 < ICC < 0.90) [50]. The only measurement that revealed poor repeatability (ICC < 0.50) was L1-MP.

### 3.2. Descriptive Statistics

Table S3 presents the descriptive statistics for the cephalometric variables at T1, T2, and T3, encompassing measures such as mean, standard deviation, minimum, and maximum. A significant increase in mandibular length was observed between T1 and T2, with an average growth of 15.11 mm. Furthermore, between T2 and T3, the mandible exhibited continued growth, with an additional 5.78 mm. In total, there was a cumulative increase of 20.89 mm in mandibular length between T1 and T3.

In comparison to mandibular length, the *y*-axis demonstrated relatively minimal changes throughout puberty. Between T1 and T2, there was an average decrease of 0.14° in the *y*-axis. Furthermore, an additional decline of 0.34° was observed between T2 and T3, resulting in a cumulative decrease of 0.48° in the *y*-axis over the entire observation period (T1–T3).

### 3.3. Prediction of the Post-Pubertal Mandibular Length

**Figure 3:**
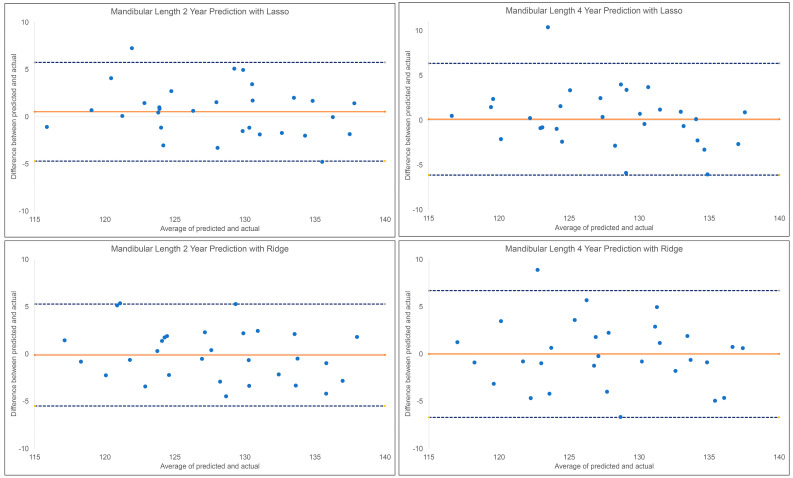
BlandAltman plots for 2 year and 4 year predictions of post-pubertal mandibular length using Lasso (**top**) and Ridge (**bottom**). The blue dashed lines represent the upper and lower bounds of the 95% confidence intervals. The orange solid line represents the mean difference between the predicted and actual mandibular length.

The results for the 2 year and 4 year predictions of post-pubertal mandibular length are shown in Table 1 and Figure 3. For the 2 year prediction, MAEs ranged from 2.11 mm to 6.07 mm, with Lasso being the most accurate and Linear Regression being the least accurate. Accuracy percentages ranged from 95.26% to 98.35% between the models employed. The Lasso, Ridge, and MLP models demonstrated an excellent correlation between predicted and actual values (0.90 < ICCs), while XGBoost, Random Forest, and SVR showed good correlations (0.75 < ICCs < 0.90). Linear Regression was the only model with a moderate correlation between the predicted and actual values (ICC: 0.58). Similarly, the 4 year prediction MAEs ranged from 2.32 mm to 5.28 mm, with Lasso being the most accurate and Linear Regression being the least accurate. All methods demonstrated a moderate to good correlation between the predicted and actual values (0.67< ICCs < 0.84). The accuracy percentages ranged from 95.88% to 98.19%.

**Figure 4:**
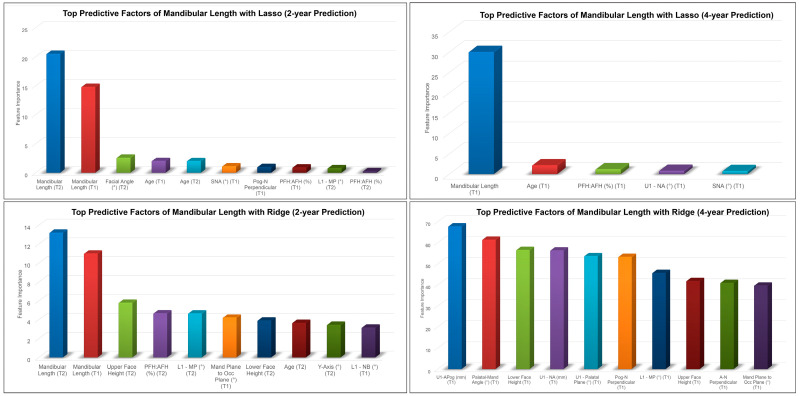
Top predictive factors for the 2 year and 4 year predictions of post-pubertal mandibular length using Lasso (**top**) and Ridge (**bottom**).

Mandibular length, age, PFH:AFH, and SNA at earlier time points were among the top predictive factors for the 2 year and 4 year predictions of post-pubertal mandibular length using Lasso (Figure 4). On the other hand, the Ridge model picked up U1 to APog distance, mandibular length, upper and lower face heights, L1-MP, and mandibular plane to occlusal plane angles as the most predictive factors of post-pubertal mandibular length.

### 3.4. Prediction of the Post-Pubertal y-Axis

**Figure 5:**
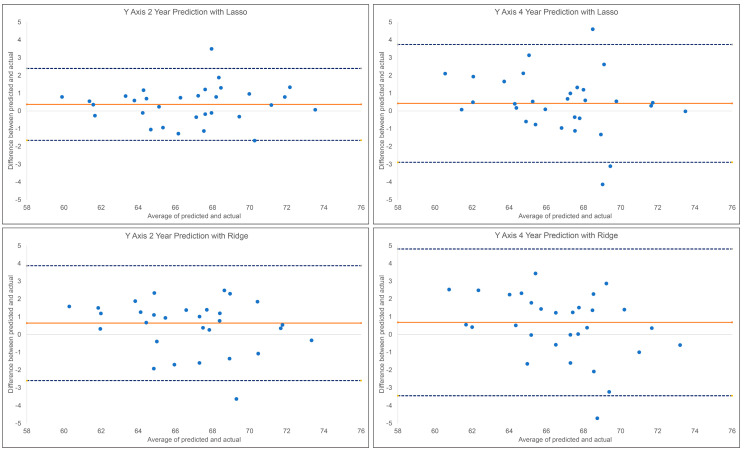
Bland–Altman plots for the 2 year and 4 year predictions of post-pubertal *y*-axis using Lasso (**top**) and Ridge (**bottom**). The blue dashed lines represent the upper and lower bounds of the 95% confidence intervals. The orange solid line represents the mean difference between the predicted and actual *y*-axis.

The results of the 2 year and 4 year predictions of the post-pubertal *y*-axis are shown in Table 2 and Figure 5. For the 2 year prediction, MAEs ranged from 0.85° to 2.74°, with Lasso being the most accurate and Linear Regression being the least accurate. Random Forest and Lasso demonstrated an excellent correlation between the predicted and actual values (0.90 < ICCs), whereas XGBoost, Ridge, SVR, and MLP showed good correlations (0.75 < ICCs < 0.90). Linear Regression was the only model with a moderate correlation between the predicted and actual values (ICC: 0.63). The accuracy percentages ranged from 96.02% to 98.76% between the models employed. For the 4 year prediction, MAEs ranged from 1.25° to 1.72°, with Lasso being the most accurate and Random Forest and SVR being the least accurate. All methods demonstrated a good correlation between the predicted and actual values (0.76< ICCs <0.86). The accuracy percentages ranged from 97.50% to 98.18%.

**Figure 6:**
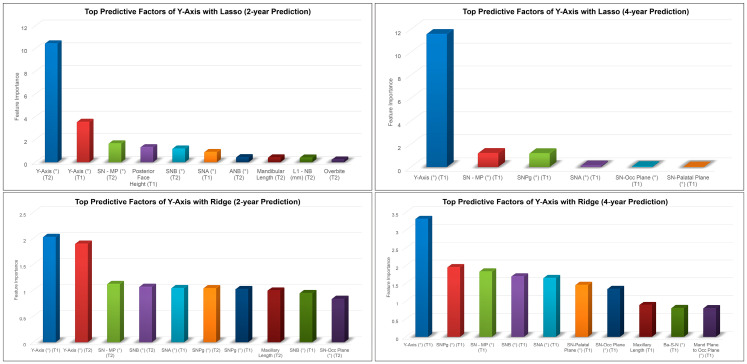
Top predictive factors for the 2 year and 4 year predictions of post-pubertal *y*-axis using Lasso (**top**) and Ridge (**bottom**).

*y*-axis, SN-MP, and SNA angles at earlier time points were among the top predictive factors for the 2 year and 4 year predictions of the post-pubertal *y*-axis using Lasso (Figure 6). In addition to these features, the Ridge model picked up SN-Pog, SNB, and SN-Occlusal Plane angles as the most predictive factors of the post-pubertal *y*-axis.

### 3.5. Method Comparison

The directional and absolute difference comparisons between the ML methods for the 2 year prediction of post-pubertal mandibular length are shown in Table 3. Linear Regression showed significantly larger absolute differences from the actual values compared to all the other methods (*p* < 0.05). Additionally, SVR exhibited significantly larger absolute differences from the actual values compared to Lasso and Ridge (*p* < 0.05). In the case of the 4 year prediction for male mandibular growth, Linear Regression demonstrated significantly larger absolute differences from the actual values compared to all the other methods, whereas Random Forest and SVR showed significantly larger absolute differences compared to Lasso (*p* < 0.05) (Table 4).

In terms of the *y*-axis prediction for the 2 year prediction, Lasso exhibited significantly smaller absolute differences from the actual values compared to Linear Regression, Random Forest, Ridge, and SVR (*p* < 0.05) (Table 5). Conversely, for the 4 year projection, Linear Regression had significantly larger absolute differences from the actual values compared to all the other methods (*p* < 0.05) (Table 6).

When comparing the prediction methods for both the 2 year and 4 year predictions of mandibular length, no significant differences were found in terms of absolute differences or directional differences for any of the methods (*p* > 0.05) (Table 7). However, when considering the *y*-axis, the absolute differences between the predicted and actual values were significantly larger when using the 2 year prediction compared to the 4 year prediction for Linear Regression (*p* < 0.001) (Table 8). Additionally, the directional differences between the predicted and actual values in the *y*-axis were significantly smaller when using the 2 year prediction compared to the 4 year prediction for Linear Regression (*p* < 0.05). Specifically, the predicted values were on average higher than the actual values for the 4 year prediction, but slightly lower on average than the actual values for the 2 year prediction. Moreover, the *y*-axis absolute differences between the predicted and actual values were significantly larger when using the 4 year prediction data compared to the 2 year prediction data for Random Forest (*p* < 0.05) (Table 8).

## 4. Discussion

There is a significant degree of variability in both the magnitude and direction of pubertal mandibular growth across different genders, races, and even among individuals of the same age and gender [51]. To thoroughly investigate the complex growth patterns of the mandible, we employed a targeted approach by selecting specific samples based on malocclusion, gender, and age. This study specifically focused on analyzing records exclusively from Class II males in the circumpubertal stage. By utilizing data from individuals aged 11 to 16 years, we were able to examine the peak growth and maturation stages that most males experience, capturing a more stable estimate of the final position of the mandible as growth approaches its plateau. Our intention was to create a novel ML model that can predict the magnitude and direction of pubertal mandibular growth in males with Class II malocclusion.

This study is a vital contribution to an extensive series of investigations utilizing advanced ML techniques to forecast the intricate process of mandibular growth. Baumrind et al. conducted a study in which orthodontists attempted to forecast the mandibular growth of Class II patients, ultimately leading to the conclusion that human predictions fare no better than chance [52]. Conversely, our study achieved an elevated level of precision by accurately predicting the post-pubertal mandibular length within a margin of 2.5 mm. In a similar vein, Wood et al. successfully predicted the mandibular length among Class I males with an accuracy within 3 mm [48]. ML exhibited an exceptional capability for accurately predicting the *y*-axis within a narrow range of 1 degree. Notably, Wood et al. also predicted the *y*-axis in a range of 1 degree [48].

Different predictors were prominent in each ML model. In terms of mandibular length, significant predictors that were identified include chronological age, upper and lower face heights, and upper incisor position. The strong predictive power of chronological age is inherently logical, given that the patients were situated within the circumpubertal age, a period characterized by accelerated growth and development. It is noteworthy that the algorithm likely detected the average peak height velocity, which typically transpires around the age of 14 years, enabling more accurate predictions [2]. Lower face height also contributed significantly to precise predictions. Hypodivergent patients with a short lower face height tend to exhibit more forward growth, whereas hyperdivergent patients with a long lower face height exhibit more vertical growth [53]. Furthermore, the position of the upper incisor plays a role in this regard. Class II Division 1 malocclusion is typified by protruded maxillary incisors, whereas Class II Division 2 patients exhibit retruded maxillary incisors. Since Class II Division 2 patients commonly have a shorter lower face height [54], the algorithm may have leveraged this information to identify them as forward growers. These predictive factors indicate that the ML algorithms were possibly capable of differentiating between Class II Division 1 and Class II Division 2 patients to discern the appropriate growth pattern more accurately.

Regarding the *y*-axis, the most predictive factors were identified as SN-MP, SN-Pog, SNB, SNA, and SN-Palatal plane. SN-MP is a measurement of the mandibular plane angle relative to the cranial base. The SN-MP angle provides the mandibular rotation model that is hypodivergent, normodivergent, or hyperdivergent. The larger the SN-MP angle, the more the mandible tends to become steeper, and the more the chin moves backward [54]. The *y*-axis is another cephalometric measurement used to assess the direction of the mandibular growth: downward and backward or downward and forward. Both the SN-MP angle and the *y*-axis angle are used to evaluate the skeletal and growth patterns in orthodontics and orthognathic surgery. They help orthodontists and surgeons understand the vertical dimensions of the face, the inclination of the mandible, and the overall skeletal relationships between the cranial base and the jaws. So, it is understandable that the vertical relationship between the mandible and the cranial base helps predict the vertical direction of growth via the *y*-axis. This is in agreement with Schudy, who found that SN-MP is closely associated with the growth and morphology of the mandible when he sought to identify the specific increments of growth responsible for the rotation of the mandible [54]. Schudy found that the larger the SN-MP angle, the more the mandible tends to become steeper, and the more the chin moves backward, and the smaller the angle, the greater the tendency of the mandible to become flatter and the chin to grow forward [54]. Additionally, the ML models utilized anterior–posterior measurements, such as SNA and SNB, to predict the *y*-axis. A larger SNB may indicate a more forward mandibular growth. By assessing these sagittal measurements, AI could make predictions about how the mandible will likely grow in relation to the rest of the face.

When comparing the ML techniques to one another, none showed a clear superiority to the others. However, Linear Regression may have performed worse than the others due to its inherent limitations. Linear Regression assumes a linear relationship between the predictor variables and the response variable, which may not accurately capture the non-linearities present in human growth patterns [55]. On the other hand, the Lasso and Ridge techniques incorporate regularization, which helps address issues of overfitting and model complexity. The Lasso performs both variable selection and regularization by imposing a penalty on the absolute values of the coefficients, effectively shrinking less important predictors to zero [55]. This feature helps in identifying the most relevant predictors for growth prediction. Ridge, on the other hand, adds a penalty term based on the square of the coefficients, which allows for a better balance between bias and variance [56]. By considering non-linear relationships and incorporating regularization techniques, Lasso and Ridge are better equipped to handle the complexities involved in predicting human growth with AI. When assessing the overall performance, the authors would consider further studies using the Lasso prediction model.

The authors acknowledge certain limitations of this current study. First, the sample size was relatively small due to the constraints of the available records in the AAOF Legacy Collection. It is worth noting that when employing ML techniques, a larger sample size is desirable as it allows for a more representative and diverse dataset. This, in turn, increases the likelihood of capturing the true underlying patterns and characteristics of the population, thereby reducing sampling bias and enhancing the model’s ability to make predictions on unseen data. Moreover, a larger sample size would help mitigate the impact of random variation and minimize instances of overfitting. Another limitation is that many images did not include sufficient facial tissue in the lateral cephalogram, which could have potentially improved the accuracy of the prediction methods. Additionally, the utilization of automated cephalometric landmark identification methods could have ensured consistency in cephalometric analyses.

## 5. Conclusions

The tested ML algorithms successfully predicted the post-pubertal mandibular length within a range of 2.5 mm and the *y*-axis within 1°. Beyond the initial mandibular length, several key predictors emerged for mandibular length, including chronological age, upper and lower face heights, and upper and lower incisor positions and inclinations. Similarly, for the *y*-axis, significant predictive factors encompassed *y*-axis measurements at earlier time points, as well as the SN-MP, SN-Pog, SNB, and SNA angles. Upon comparing the prediction methods for both the 2 year and 4 year forecasts of mandibular length, no substantial differences surfaced in terms of absolute disparities or directional variations among any of the methods. However, regarding the *y*-axis, employing the 2-year prediction resulted in significantly larger absolute deviations between the predicted and actual values compared to the 4 year prediction when utilizing Linear Regression. While the potential of ML techniques to accurately anticipate future mandibular growth in Class II cases holds promise, further research is imperative. Larger sample sizes and more extensive data points are needed to refine the precision of these predictions.

## Figures and Tables

**Table 1:** Results of the 2 year and 4 year prediction of post-pubertal mandibular length.

	2 Year Prediction					4 Year Prediction				
Models	MAE	RMSE	ME	ICC	Accuracy %	MAE	RMSE	ME	ICC	Accuracy %
XGBoost	2.80	3.29	−0.42	0.88	97.81	3.20	3.97	0.02	0.81	97.50
Random Forest	2.83	3.58	−0.56	0.83	97.79	3.55	4.39	0.34	0.71	97.23
Lasso	2.11	2.68	−0.54	0.91	98.35	2.32	3.13	−0.12	0.87	98.19
Ridge	2.29	2.71	0.10	0.91	98.21	2.62	3.37	−0.01	0.87	97.95
Linear Regression	6.07	7.65	2.32	0.58	95.26	5.28	6.33	0.84	0.67	95.88
SVR	3.55	4.14	−0.29	0.78	97.23	3.41	4.01	−0.70	0.74	97.33
MLP	2.65	3.08	−0.46	0.90	97.93	3.09	3.90	−1.74	0.84	97.59

MAE: Mean absolute error, RMSE: root mean square error, ME: mean error, and ICC: intra-class coefficient.

**Table 2:** Results of the 2 year and 4 year prediction of the post-pubertal *y*-axis.

	2 Year Prediction					4 Year Prediction				
Models	MAE	RMSE	ME	ICC	Accuracy %	MAE	RMSE	ME	ICC	Accuracy %
XGBoost	1.20	1.55	−0.87	0.89	98.26	1.52	1.93	−0.62	0.82	97.79
Random Forest	1.17	1.45	−0.66	0.90	98.30	1.72	2.10	−0.79	0.77	97.50
Lasso	0.85	1.08	−0.37	0.95	98.76	1.25	1.72	−0.42	0.86	98.18
Ridge	1.41	1.74	−0.64	0.86	97.95	1.68	2.18	−0.68	0.77	97.56
Linear Regression	2.74	3.78	0.32	0.63	96.02	1.66	2.24	−0.62	0.81	97.59
SVR	1.31	1.75	−0.39	0.86	98.10	1.72	2.25	−0.45	0.76	97.50
MLP	1.30	1.62	0.00	0.89	98.11	1.49	1.91	−0.38	0.83	97.83

MAE: Mean absolute error, RMSE: root mean square error, ME: mean error, and ICCL intra-class correlation coefficient.

**Table 3:** Directional and absolute difference comparisons between ML models for the 2 year prediction of post-pubertal mandibular length.

Directional Difference		Absolute Difference	
Result	*p*-Value	Result	*p*-Value
Lasso > Linear Regression	<0.001	Lasso < Linear Regression	<0.001
Lasso and MLP	0.922	Lasso and MLP	0.336
Lasso and Random Forest	0.971	Lasso and Random Forest	0.196
Lasso and Ridge	0.407	Lasso and Ridge	0.752
Lasso and SVR	0.745	Lasso < SVR	0.010
Lasso and XGBoost	0.882	Lasso and XGBoost	0.213
Linear Regression < MLP	<0.001	Linear Regression > MLP	<0.001
Linear Regression < Random Forest	<0.001	Linear Regression > Random Forest	<0.001
Linear Regression < Ridge	0.004	Linear Regression > Ridge	<0.001
Linear Regression < SVR	0.001	Linear Regression > SVR	<0.001
Linear Regression < XGBoost	<0.001	Linear Regression > XGBoost	<0.001
MLP and Random Forest	0.893	MLP and Random Forest	0.739
MLP and Ridge	0.464	MLP and Ridge	0.517
MLP and SVR	0.820	MLP and SVR	0.105
MLP and XGBoost	0.959	MLP and XGBoost	0.777
Random Forest and Ridge	0.386	Random Forest and Ridge	0.327
Random Forest and SVR	0.717	Random Forest and SVR	0.197
Random Forest and XGBoost	0.853	Random Forest and XGBoost	0.961
Ridge and SVR	0.614	Ridge < SVR	0.024
Ridge and XGBoost	0.496	Ridge and XGBoost	0.352
SVR and XGBoost	0.860	SVR and XGBoost	0.181

**Table 4:** Directional and absolute difference comparisons between ML models for the 4 year prediction of post-pubertal mandibular length.

Directional Difference		Absolute Difference	
Result	*p*-Value	Result	*p*-Value
Lasso and Linear Regression	0.171	Lasso < Linear Regression	<0.001
Lasso < MLP	0.021	Lasso and MLP	0.115
Lasso and Random Forest	0.508	Lasso < Random Forest	0.013
Lasso and Ridge	0.876	Lasso and Ridge	0.536
Lasso and SVR	0.405	Lasso < SVR	0.027
Lasso and XGBoost	0.846	Lasso and XGBoost	0.075
Linear Regression < MLP	<0.001	Linear Regression > MLP	<0.001
Linear Regression and Random Forest	0.478	Linear Regression > Random Forest	0.001
Linear Regression and Ridge	0.225	Linear Regression > Ridge	<0.001
Linear Regression < SVR	0.028	Linear Regression > SVR	<0.001
Linear Regression and XGBoost	0.239	Linear Regression > XGBoost	<0.001
MLP > Random Forest	0.003	MLP and Random Forest	0.357
MLP > Ridge	0.014	MLP and Ridge	0.337
MLP and SVR	0.137	MLP and SVR	0.518
MLP > XGBoost	0.013	MLP and XGBoost	0.834
Random Forest and Ridge	0.613	Random Forest and Ridge	0.061
Random Forest and SVR	0.136	Random Forest and SVR	0.783
Random Forest and XGBoost	0.639	Random Forest and XGBoost	0.476
Ridge and SVR	0.323	Ridge and SVR	0.109
Ridge and XGBoost	0.970	Ridge and XGBoost	0.243
SVR and XGBoost	0.305	SVR and XGBoost	0.662

**Table 5:** Directional and absolute difference comparisons between ML methods for the 2 year prediction of the *y*-axis.

Directional Difference		Absolute Difference	
Result	*p*-Value	Result	*p*-Value
Lasso and Linear Regression	0.096	Lasso < Linear Regression	<0.001
Lasso and MLP	0.374	Lasso and MLP	0.121
Lasso and Random Forest	0.483	Lasso and Random Forest	0.270
Lasso and Ridge	0.514	Lasso and Ridge	0.052
Lasso and SVR	0.968	Lasso and SVR	0.109
Lasso and XGBoost	0.229	Lasso and XGBoost	0.228
Linear Regression and MLP	0.434	Linear Regression > MLP	<0.001
Linear Regression < Random Forest	0.018	Linear Regression > Random Forest	<0.001
Linear Regression < Ridge	0.021	Linear Regression > Ridge	<0.001
Linear Regression and SVR	0.088	Linear Regression > SVR	<0.001
Linear Regression < XGBoost	0.004	Linear Regression > XGBoost	<0.001
MLP and Random Forest	0.113	MLP and Random Forest	0.651
MLP and Ridge	0.124	MLP and Ridge	0.692
MLP and SVR	0.353	MLP and SVR	0.960
MLP < XGBoost	0.037	MLP and XGBoost	0.727
Random Forest and Ridge	0.961	Random Forest and Ridge	0.396
Random Forest and SVR	0.508	Random Forest and SVR	0.615
Random Forest and XGBoost	0.614	Random Forest and XGBoost	0.917
Ridge and SVR	0.540	Ridge and SVR	0.729
Ridge and XGBoost	0.580	Ridge and XGBoost	0.456
SVR and XGBoost	0.244	SVR and XGBoost	0.690

**Table 6:** Directional and absolute difference comparisons between the ML models for the 4 year prediction of the *y*-axis.

Directional Difference		Absolute Difference	
Result	*p*-Value	Result	*p*-Value
Lasso and Linear Regression	0.337	Lasso < Linear Regression	0.013
Lasso and MLP	0.823	Lasso and MLP	0.133
Lasso and Random Forest	0.070	Lasso < Random Forest	0.004
Lasso and Ridge	0.203	Lasso < Ridge	0.008
Lasso and SVR	0.891	Lasso < SVR	0.004
Lasso and XGBoost	0.339	Lasso and XGBoost	0.093
Linear Regression and MLP	0.237	Linear Regression and MLP	0.314
Linear Regression and Random Forest	0.390	Linear Regression and Random Forest	0.705
Linear Regression and Ridge	0.753	Linear Regression and Ridge	0.880
Linear Regression and SVR	0.411	Linear Regression and SVR	0.721
Linear Regression and XGBoost	0.997	Linear Regression and XGBoost	0.407
MLP < Random Forest	0.042	MLP and Random Forest	0.167
MLP and Ridge	0.135	MLP and Ridge	0.247
MLP and SVR	0.718	MLP and SVR	0.173
MLP and XGBoost	0.239	MLP and XGBoost	0.858
Random Forest and Ridge	0.585	Random Forest and Ridge	0.820
Random Forest and SVR	0.093	Random Forest and SVR	0.983
Random Forest and XGBoost	0.387	Random Forest and XGBoost	0.228
Ridge and SVR	0.256	Ridge and SVR	0.837
Ridge and XGBoost	0.750	Ridge and XGBoost	0.327
SVR and XGBoost	0.413	SVR and XGBoost	0.236

**Table 7:** Comparisons of the directional and absolute differences between the 2 year and 4 year predictions of post-pubertal mandibular length.

	Directional Difference		Absolute Difference	
Method	Result	*p*-Value	Result	*p*-Value
XGBoost	2-year and 4-year	0.577	2-year and 4-year	0.495
Random Forest	2-year and 4-year	0.250	2-year and 4-year	0.213
Lasso	2-year and 4-year	0.595	2-year and 4-year	0.724
Ridge	2-year and 4-year	0.889	2-year and 4-year	0.563
Linear Regression	2-year and 4-year	0.062	2-year and 4-year	0.169
SVR	2-year and 4-year	0.603	2-year and 4-year	0.811
MLP	2-year and 4-year	0.107	2-year and 4-year	0.437

**Table 8:** Comparisons of the directional and absolute differences between the 2 year and 4 year predictions of the post-pubertal *y*-axis.

	Directional Difference		Absolute Difference	
Method	Result	*p*-Value	Result	*p*-Value
XGBoost	2-year and 4-year	0.500	2-year and 4-year	0.214
Random Forest	2-year and 4-year	0.724	2-year < 4-year	0.036
Lasso	2-year and 4-year	0.886	2-year and 4-year	0.126
Ridge	2-year and 4-year	0.912	2-year and 4-year	0.305
Linear Regression	2-year < 4-year	0.012	2-year > 4-year	<0.001
SVR	2-year and 4-year	0.863	2-year and 4-year	0.125
MLP	2-year and 4-year	0.314	2-year and 4-year	0.455

## Data Availability

The data underlying this article are available in the article. The datasets were obtained from sources in the public domain from the AAOF Legacy Collection at https://www.aaoflegacycollection.org/ (accessed on 18 August 2023).

## Supplementary Materials

The following supporting information can be downloaded at: https://www.mdpi.com/article/10.3390/diagnostics13162713/s1, Table S1: Cephalometric variables and their definitions. Table S2: Intra-examiner repeatability of the measurements. Table S3: The descriptive statistics of the cephalometric measurements at T1, T2, and T3, including mean, standard deviation, and minimum/maximum values.

## References

[1] Manlove A.E., Romeo G., Venugopalan S.R. (2020). Craniofacial growth: Current theories and influence on management. Oral. Maxillofac. Surg. Clin. N. Am..

[2] Tsutsui T., Iizuka S., Sakamaki W., Maemichi T., Torii S. (2022). Growth until Peak Height Velocity Occurs Rapidly in Early Maturing Adolescent Boys. Children.

[3] McNamara J.A. (1981). Components of class II malocclusion in children 8–10 years of age. Angle Orthod..

[4] Sayin M.O., Türkkahraman H. (2005). Cephalometric evaluation of nongrowing females with skeletal and dental Class II, division 1 malocclusion. Angle Orthod..

[5] Stahl F., Baccetti T., Franchi L., McNamara J.A. (2008). Longitudinal growth changes in untreated subjects with Class II Division 1 malocclusion. Am. J. Orthod. Dentofacial Orthop..

[6] Türkkahraman H., Sayin M.O. (2006). Effects of activator and activator headgear treatment: Comparison with untreated Class II subjects. Eur. J. Orthod..

[7] Bjork A. (1963). Variations in the growth pattern of the human mandible: Longitudinal radiographic study by the implant method. J. Dent. Res..

[8] Björk A. (1968). The use of metallic implants in the study of facial growth in children: Method and application. Am. J. Phys. Anthropol..

[9] Björk A. (1969). Prediction of mandibular growth rotation. Am. J. Orthod..

[10] Skieller V., Björk A., Linde-Hansen T. (1984). Prediction of mandibular growth rotation evaluated from a longitudinal implant sample. Am. J. Orthod..

[11] Leslie L.R., Southard T.E., Southard K.A., Casko J.S., Jakobsen J.R., Tolley E.A., Hillis S.L., Carolan C., Logue M. (1998). Prediction of mandibular growth rotation: Assessment of the Skieller, Björk, and Linde-Hansen method. Am. J. Orthod. Dentofac. Orthop..

[12] Ricketts R.M. (1972). A principle of arcial growth of the mandible. Angle Orthod..

[13] Mitchell D.L., Jordan J.F., Ricketts R.M. (1975). Arcial growth with metallic implants in mandibular growth prediction. Am. J. Orthod..

[14] Rudolph D.J., White S.E., Sinclair P.M. (1998). Multivariate prediction of skeletal Class II growth. Am. J. Orthod. Dentofacial Orthop..

[15] Buschang P., Tanguay R., LaPalme L., Demirjian A. (1990). Mandibular growth prediction: Mean growth increments versus mathematical models. Eur. J. Orthod..

[16] Jiménez-Silva A., Carnevali-Arellano R., Vivanco-Coke S., Tobar-Reyes J., Araya-Díaz P., Palomino-Montenegro H. (2021). Craniofacial growth predictors for class II and III malocclusions: A systematic review. Clin. Exp. Dent. Res..

[17] Walker G.F. (1972). A new approach to the analysis of craniofacial morphology and growth. Am. J. Orthod..

[18] Vilone G., Longo L. (2020). Explainable artificial intelligence: A systematic review. arXiv.

[19] Rajkomar A., Dean J., Kohane I. (2019). Machine learning in medicine. N. Engl. J. Med..

[20] Panesar S., Zhao A., Hollensbe E., Wong A., Bhamidipalli S.S., Eckert G., Dutra V., Turkkahraman H. (2023). Precision and Accuracy Assessment of Cephalometric Analyses Performed by Deep Learning Artificial Intelligence with and without Human Augmentation. Appl. Sci..

[21] Bulatova G., Kusnoto B., Grace V., Tsay T.P., Avenetti D.M., Sanchez F.J.C. (2021). Assessment of automatic cephalometric landmark identification using artificial intelligence. Orthod. Craniofac Res..

[22] Kim J., Kim I., Kim Y.J., Kim M., Cho J.H., Hong M., Kang K.H., Lim S.H., Kim S.J., Kim Y.H. (2021). Accuracy of automated identification of lateral cephalometric landmarks using cascade convolutional neural networks on lateral cephalograms from nationwide multi-centres. Orthod. Craniofac Res..

[23] Lindner C., Wang C.W., Huang C.T., Li C.H., Chang S.W., Cootes T.F. (2016). Fully Automatic System for Accurate Localisation and Analysis of Cephalometric Landmarks in Lateral Cephalograms. Sci. Rep..

[24] Popova T., Stocker T., Khazaei Y., Malenova Y., Wichelhaus A., Sabbagh H. (2023). Influence of growth structures and fixed appliances on automated cephalometric landmark recognition with a customized convolutional neural network. BMC Oral. Health.

[25] Xie X., Wang L., Wang A. (2010). Artificial neural network modeling for deciding if extractions are necessary prior to orthodontic treatment. Angle Orthod..

[26] Suhail Y., Upadhyay M., Chhibber A., Kshitiz (2020). Machine Learning for the Diagnosis of Orthodontic Extractions: A Computational Analysis Using Ensemble Learning. Bioengineering.

[27] Ryu J., Kim Y.H., Kim T.W., Jung S.K. (2023). Evaluation of artificial intelligence model for crowding categorization and extraction diagnosis using intraoral photographs. Sci. Rep..

[28] Real A.D., Real O.D., Sardina S., Oyonarte R. (2022). Use of automated artificial intelligence to predict the need for orthodontic extractions. Korean J. Orthod..

[29] Mason T., Kelly K.M., Eckert G., Dean J.A., Dundar M.M., Turkkahraman H. (2023). A machine learning model for orthodontic extraction/non-extraction decision in a racially and ethnically diverse patient population. Int. Orthod..

[30] Li P., Kong D., Tang T., Su D., Yang P., Wang H., Zhao Z., Liu Y. (2019). Orthodontic Treatment Planning based on Artificial Neural Networks. Sci. Rep..

[31] Kapoor S., Shyagali T.R., Kuraria A., Gupta A., Tiwari A., Goyal P. (2023). An artificial neural network approach for rational decision-making in borderline orthodontic cases: A preliminary analytical observational in silico study. J. Orthod..

[32] Jung S.K., Kim T.W. (2016). New approach for the diagnosis of extractions with neural network machine learning. Am. J. Orthod. Dentofacial Orthop..

[33] Etemad L., Wu T.H., Heiner P., Liu J., Lee S., Chao W.L., Zaytoun M.L., Guez C., Lin F.C., Jackson C.B. (2021). Machine learning from clinical data sets of a contemporary decision for orthodontic tooth extraction. Orthod. Craniofac Res..

[34] Leavitt L., Volovic J., Steinhauer L., Mason T., Eckert G., Dean J.A., Dundar M.M., Turkkahraman H. (2023). Can we predict orthodontic extraction patterns by using machine learning?. Orthod. Craniofac Res..

[35] Lee H., Ahmad S., Frazier M., Dundar M.M., Turkkahraman H. (2022). A novel machine learning model for class III surgery decision. J. Orofac. Orthop./Fortschritte Kieferorthopädie.

[36] Radwan M.T., Sin Ç., Akkaya N., Vahdettin L. (2022). Artificial intelligence-based algorithm for cervical vertebrae maturation stage assessment. Orthod. Craniofac Res..

[37] Mohammad-Rahimi H., Motamadian S.R., Nadimi M., Hassanzadeh-Samani S., Minabi M.A.S., Mahmoudinia E., Lee V.Y., Rohban M.H. (2022). Deep learning for the classification of cervical maturation degree and pubertal growth spurts: A pilot study. Korean J. Orthod..

[38] Liao N., Dai J., Tang Y., Zhong Q., Mo S. (2022). iCVM: An Interpretable Deep Learning Model for CVM Assessment Under Label Uncertainty. IEEE J. Biomed. Health Inform..

[39] Li H., Chen Y., Wang Q., Gong X., Lei Y., Tian J., Gao X. (2022). Convolutional neural network-based automatic cervical vertebral maturation classification method. Dentomaxillofac Radiol..

[40] Kök H., Izgi M.S., Acilar A.M. (2021). Determination of growth and development periods in orthodontics with artificial neural network. Orthod. Craniofac Res..

[41] Kim D.W., Kim J., Kim T., Kim T., Kim Y.J., Song I.S., Ahn B., Choo J., Lee D.Y. (2021). Prediction of hand-wrist maturation stages based on cervical vertebrae images using artificial intelligence. Orthod. Craniofac Res..

[42] Atici S.F., Ansari R., Allareddy V., Suhaym O., Cetin A.E., Elnagar M.H. (2023). AggregateNet: A deep learning model for automated classification of cervical vertebrae maturation stages. Orthod. Craniofac Res..

[43] Atici S.F., Ansari R., Allareddy V., Suhaym O., Cetin A.E., Elnagar M.H. (2022). Fully automated determination of the cervical vertebrae maturation stages using deep learning with directional filters. PLoS ONE.

[44] Amasya H., Yildirim D., Aydogan T., Kemaloglu N., Orhan K. (2020). Cervical vertebral maturation assessment on lateral cephalometric radiographs using artificial intelligence: Comparison of machine learning classifier models. Dentomaxillofac Radiol..

[45] Niño-Sandoval T.C., Pérez S.V.G., González F.A., Jaque R.A., Clementina Infante-Contreras (2017). Use of automated learning techniques for predicting mandibular morphology in skeletal class I, II and III. Forensic Sci. Int..

[46] Niño-Sandoval T.C., Perez S.V.G., Gonzalez F.A., Jaque R.A., Clementina Infante-Contreras (2016). An automatic method for skeletal patterns classification using craniomaxillary variables on a Colombian population. Forensic Sci. Int..

[47] Jiwa S. (2020). Applicability of Deep Learning for Mandibular Growth Prediction.

[48] Wood T., Anigbo J.O., Eckert G., Stewart K.T., Dundar M.M., Turkkahraman H. (2023). Prediction of the Post-Pubertal Mandibular Length and Y Axis of Growth by Using Various Machine Learning Techniques: A Retrospective Longitudinal Study. Diagnostics.

[49] AAOF Craniofacial Growth Legacy Collection. https://www.aaoflegacycollection.org/aaof_home.html.

[50] Koo T.K., Li M.Y. (2016). A guideline of selecting and reporting intraclass correlation coefficients for reliability research. J. Chiropr. Med..

[51] Ochoa B.K., Nanda R.S. (2004). Comparison of maxillary and mandibular growth. Am. J. Orthod. Dentofacial Orthop..

[52] Baumrind S., Korn E.L., West E.E. (1984). Prediction of mandibular rotation: An empirical test of clinician performance. Am. J. Orthod..

[53] Karlsen A.T. (1995). Craniofacial growth differences between low and high MP-SN angle males: A longitudinal study. Angle Orthod..

[54] Schudy F.F. (1965). The rotation of the mandible resulting from growth: Its implications in orthodontic treatment. Angle Orthod..

[55] Su X., Yan X., Tsai C.L. (2012). Linear regression. Wiley Interdiscip. Rev. Comput. Stat..

[56] McDonald G.C. (2009). Ridge regression. Wiley Interdiscip. Rev. Comput. Stat..

