# Supplementary material for: Prediction of Pubertal Mandibular Growth in Males with Class II Malocclusion by Utilizing Machine Learning
Source: Diagnostics (Basel). 2023 Aug 21;13(16):2713. doi: 10.3390/diagnostics13162713 (PMC10453460; doi:10.3390/diagnostics13162713)
Supplement: Supplementary file 1 [file diagnostics-13-02713-s001.zip › diagnostics-2514202-supplementary.pdf]

Supplementary Table S1. Cephalometric variables and their definitions.

Category	Measurements	Definitions
Maxilla to Cranial Base	SNA (˚)	The angle formed by connecting sella, nasion, and A-point
	SN-Palatal Plane (˚)	The angle formed from the intersection of sella-nasion line and a line drawn connecting anterior nasal spine to posterior nasal spine
	SN-Occlusal Plane (˚)	The angle formed from sella-nasion and occlusal plane
	A-N Perpendicular (mm)	The linear distance from A point to the nasion perpendicular.
Mandible to Cranial Base	SNB (˚)	The angle formed by connecting sella, nasion, and B-point
	SNPog (˚)	The angle formed by connecting sella, naison, and pogonion
	FMA: MP-FH (˚)	The angle formed from the intersection of porion-orbitale line and a line drawn connecting gonion to gnathion
	SN-MP (˚)	The angle formed from the intersection of sella-nasion line and a line drawn connecting gonion to gnathion
	Mandibular Plane to Occlusal Plane (˚)	The angle formed by mandibular plane and occlusal plane
	B-N Perpendicular (mm)	The linear distance from B point to nasion perpendicular
	Pog-N Perpendicular (mm)	The linear distance from pogonion to nasion perpendicular
	Y-Axis: SGn-SN (˚)	The angle formed by connecting nasion, sella, and gnathion
Maxilla to Mandible	ANB (˚)	The difference between SNA and SNB
	Palatal-Mandibular Angle (PP-MP) (˚)	The angle formed from palatal plane and mandibular plane
	Wits Appraisal (mm)	The distance between A point to occlusal plane and B point to occlusal plane
	Maxillary Length: ANS-PNS (mm)	The linear measurement between anterior nasal spine and posterior nasal spine
	Mandibular Length: Co-Gn (mm)	The linear measurement between condylion and gnathion
Cranial Base	Cranial Base Flexure Angle: Ba-S-N (˚)	The angle formed by connecting basion, sella, and nasion
Upper Incisors to Maxilla	U1-SN (˚)	The angle formed from a line connecting sella to nasion and a line connecting the upper incisor incisal tip to the root apex
	U1-NA (˚)	The angle formed from a line connecting nasion to A-point and a line connecting the upper incisor incisal tip to the root apex
	U1-NA (mm)	The linear measurement from the labial surface of the upper incisor to the line connecting nasion to A-point
	U1-Palatal Plane (˚)	The angle formed by the position of maxillary incisor to palatal plane
	U1 Protrusion (U1-APog) (mm)	The distance from maxillary incisor to the A point-pogonion reference line
Lower Incisors to Mandible	L1-MP (˚)	The angle formed from a line connecting the lower incisor incisal tip to the root apex and a line connecting gonion to gnathion
	L1-NB (˚)	The angle formed from a line connecting the lower incisor incisal tip to the root apex and a line connecting nasion to B-point
	L1-NB (mm)	The linear measurement from the labial surface of the lower incisor incisal to the line connecting nasion to B-point
	L1 Protrusion (L1-APog) (mm)	The distance from mandibular incisor to the A point-pogonion reference line
Incisors to Each Other	Interincisal Angle (˚)	The angle formed from a line connecting the lower incisor incisal tip to the apex and a line connecting the upper incisor incisal tip to the root apex
	Overjet (mm)	The horizontal distance from maxillary incisor tip to mandibular incisor tip
	Overbite (mm)	The vertical distance from maxillary incisor tip to mandibular incisor tip

	Hard Tissue Upper Face Height: N-ANS (mm)	The linear measurement between nasion and anterior nasal spine
	Hard Tissue Lower Face Height: ANS-Me (mm)	The linear measurement between anterior nasal spine and menton
	UFH (N-ANS/(N-ANS+ANS-Me)) (%)	The ratio of the upper face height to facial height
	LFH (ANS-Me/(N-ANS+ANS-Me)) (%)	The ratio of lower face heigh to facial height
	Posterior Face Height: Ar-Go (mm)	The linear measurement between articulare and gonion
	PFH:AFH (Co-Go:N-Me) (%)	The ratio of posterior facial height to anterior facial height
Profile	Convexity: NA-APog (°)	The angle formed by connecting nasion, A-point, and pogonion
	Facial Angle: FH-NPog (°)	The angle formed from a line connecting porion to orbitale and a line connecting nasion to pogonion

Supplementary Table S2. Intra-examiner repeatability of the measurements.

Measurements	Mean	SD	P-value	ICC
SNA (°)	0.06	0.77	0.81	0.96
SN-Palatal Plane (°)	-0.70	0.70	0.01	0.95
SN-Occlusal Plane (°)	-0.32	1.93	0.61	0.73
A-N Perpendicular (mm)	0.15	1.72	0.79	0.93
SNB (°)	0.11	0.73	0.65	0.95
SNPog (°)	0.07	0.96	0.82	0.89
FMA: MP-FH (°)	-0.01	0.99	0.98	0.98
SN – MP (°)	-0.36	1.58	0.49	0.94
Mandibular Plane to Occlusal Plane (°)	0.33	1.86	0.59	0.88
B-N Perpendicular (mm)	-0.12	2.57	0.89	0.84
Pog-N Perpendicular (mm)	-0.24	2.75	0.79	0.91
Y-Axis (SGn-SN) (°)	-0.14	0.56	0.45	0.98
ANB (°)	-0.05	0.93	0.87	0.81
Palatal-Mandibular Angle (PP-MP) (°)	0.72	1.28	0.11	0.97
Wits Appraisal (mm)	0.29	1.69	0.60	0.90
Maxillary length (ANS-PNS) (mm)	0.79	2.40	0.33	0.96
Mandibular length (Co-Gn) (mm)	-1.90	4.80	0.24	0.97
Cranial Base Flexure Angle: Ba-S-N (°)	0.53	1.11	0.17	0.97
U1 – SN (°)	1.45	2.31	0.08	0.96
U1 – NA (°)	1.39	2.70	0.14	0.94
U1 - NA (mm)	0.22	1.22	0.58	0.90
U1 - Palatal Plane (°)	0.75	2.07	0.28	0.98
U1 Protrusion (U1-APog) (mm)	0.25	0.67	0.27	0.98
L1 – MP (°)	2.01	3.90	0.14	0.38
L1 – NB (°)	1.85	4.18	0.20	0.78
L1 - NB (mm)	0.44	0.65	0.06	0.96
L1 Protrusion (L1-APog) (mm)	0.50	0.71	0.05	0.97
Interincisal Angle (°)	-2.71	5.35	0.14	0.91
Overjet (mm)	-0.26	0.44	0.09	0.99

Overbite (mm)	-0.04	1.09	0.91	0.86
Hard Tissue Upper Face Height (N-ANS) (mm)	-0.73	1.37	0.13	0.99
Hard Tissue ower Face Height (ANS-Me) (mm)	-0.97	2.00	0.16	0.99
UFH (N-ANS/(N-ANS+ANS-Me)) (%)	0.08	0.60	0.68	0.88
LFH (ANS-Me/(N-ANS+ANS-Me)) (%)	-0.08	0.60	0.68	0.88
Posterior Face Height (Ar-Go) (mm)	-0.81	2.34	0.30	0.94
PFH:AFH (Co-Go : N-Me) (%)	-0.43	3.20	0.68	0.61
Convexity (NA-APog) (˚)	0.06	2.50	0.94	0.81
Facial Angle (FH-NPog) (˚)	-0.19	1.28	0.65	0.90

Supplementary Table S3. The descriptive statistics of the cephalometric measurements at T1, T2, and T3, including mean, standard deviation, and minimum/maximum values.

	**T1**				**T2**				**T3**			
**Measurements**	**Mean**	**SD**	**Min**	**Max**	**Mean**	**SD**	**Min**	**Max**	**Mean**	**SD**	**Min**	**Max**
SNA (°)	81.50	3.42	73.50	89.50	81.98	3.38	75.20	91.50	82.26	3.52	73.60	93.10
SN-Palatal Plane (°)	6.81	3.39	-2.40	14.50	6.72	3.34	-2.40	14.70	6.47	3.45	-1.60	14.50
SN to Occlusal Plane (°)	16.31	3.92	6.70	25.10	14.60	4.26	4.00	26.40	13.51	4.18	1.90	26.20
A-N Perpendicular (mm)	-1.20	3.69	-15.60	7.70	-1.17	3.75	-12.20	9.90	-1.09	4.35	-14.10	10.70
SNB (°)	77.30	3.11	69.80	84.40	78.09	3.15	70.60	85.30	78.77	3.33	70.40	87.20
SNPog (°)	78.48	3.18	70.80	85.40	79.50	3.31	71.10	88.10	80.45	3.47	71.20	89.80
FMA: MP-FH (˚)	26.73	4.84	16.80	42.60	26.31	4.94	14.20	40.60	25.45	5.24	12.70	38.20
SN – MP (°)	31.70	4.74	21.40	42.00	30.81	5.00	20.80	42.50	29.81	5.07	17.70	41.80
Mandibular Plane to Occlusal Plane (°)	16.37	3.70	8.40	25.90	17.06	4.08	6.70	29.50	17.08	4.34	4.50	28.50
B-N Perpendicular (mm)	-8.84	5.51	-27.40	4.60	-8.56	5.79	-24.40	10.60	-7.93	6.85	-23.10	10.20
Pog-N Perpendicular (mm)	-7.98	6.47	-29.20	6.80	-7.15	6.89	-25.50	14.50	-5.80	8.10	-24.20	16.90
Y-Axis (SGn-SN) (°)	67.32	3.34	59.90	75.00	67.18	3.50	56.60	75.40	66.84	3.60	55.00	75.30
ANB (°)	4.19	1.92	-0.80	11.10	3.89	1.85	-0.30	8.50	3.48	2.02	-1.00	8.00
Palatal-Mandibular Angle (PP-MP) (°)	25.87	5.06	14.80	41.70	24.94	5.06	14.20	39.80	24.10	5.04	12.20	40.50
Wits Appraisal (mm)	1.82	2.57	-5.00	8.50	2.18	2.71	-5.30	10.10	2.16	2.73	-5.30	9.20
Maxillary length (ANS-PNS) (mm)	52.28	3.33	41.80	59.90	54.36	3.65	45.90	64.80	56.39	3.61	48.60	68.40
Mandibular length (Co-Gn) (mm)	107.19	6.26	89.90	124.20	122.30	7.12	101.10	139.90	128.08	7.65	105.70	147.40
Cranial Base Flexure Angle: Ba-S-N (°)	128.49	5.19	110.80	141.30	128.51	5.15	115.90	138.80	128.14	5.32	113.40	140.00
U1 – SN (°)	103.24	8.35	83.30	124.30	103.23	8.67	79.60	123.40	103.19	8.88	76.30	122.40
U1 – NA (°)	21.74	8.43	2.40	43.80	21.25	8.88	0.50	45.30	20.94	8.77	-0.50	43.30
U1 - NA (mm)	3.96	2.82	-2.30	12.30	4.20	2.98	-1.80	12.30	4.36	3.11	-2.80	13.50
U1 - Palatal Plane (°)	110.05	7.31	92.90	133.80	109.95	7.95	90.50	131.20	109.67	7.97	86.90	132.90
U1 Protrusion (U1-APog) (mm)	6.49	2.90	-3.80	14.40	6.34	3.01	-3.90	12.50	5.96	3.10	-4.80	13.00

L1 – MP (°)	94.86	6.06	78.00	112.20	94.97	5.90	78.10	111.70	94.70	6.33	75.10	111.50
L1 – NB (°)	26.16	6.13	4.30	39.70	26.24	6.34	6.00	38.40	25.62	6.68	3.00	41.00
L1 - NB (mm)	4.60	2.11	-2.30	10.00	4.85	2.28	-1.70	10.20	4.93	2.46	-2.90	11.20
L1 Protrusion (L1-APog) (mm)	1.17	2.30	-6.40	6.80	1.28	2.55	-6.70	7.20	1.29	2.69	-8.80	7.10
Interincisal Angle (°)	127.90	11.11	106.30	167.90	128.62	12.28	106.40	165.60	129.94	12.15	108.80	172.00
Overjet (mm)	5.42	2.40	1.20	13.30	5.14	2.13	0.80	12.80	4.80	2.09	0.10	11.10
Overbite (mm)	3.15	2.06	-5.20	7.10	3.12	2.13	-6.60	7.60	2.97	2.27	-5.10	7.40
Hard Tissue Upper Face Height (N-ANS) (mm)	51.52	3.42	42.50	59.40	54.12	3.61	46.60	64.20	55.98	3.80	47.20	65.20
Hard Tissue Lower Face Height (ANS-Me) (mm)	64.19	4.98	53.70	85.20	67.68	5.27	59.00	89.20	70.82	5.47	60.60	94.60
UFH (N-ANS/(N-ANS+ANS-Me)) (%)	44.57	1.98	40.40	50.00	44.46	1.90	39.40	48.70	44.18	1.98	39.20	48.30
LFH (ANS-Me/(N-ANS+ANS-Me)) (%)	55.50	2.04	50.00	61.40	55.54	1.90	51.30	60.60	55.82	1.98	51.70	60.80
Posterior Face Height (Ar-Go) (mm)	42.83	3.91	33.20	53.40	46.28	4.66	34.00	56.50	49.63	5.41	35.30	61.70
PFH:AFH (Co-Go : N-Me) (%)	54.75	3.53	45.40	62.70	55.35	4.05	45.30	66.10	56.48	3.74	48.10	66.60
Convexity (NA-APog) (°)	6.47	4.69	-7.90	22.50	5.22	4.68	-7.50	17.70	3.80	4.89	-6.10	17.20
Facial Angle (FH-NPog) (°)	85.70	3.47	74.00	93.90	86.38	3.53	76.00	97.20	87.15	4.04	77.00	98.40
